# New species of *Teratolytta* Semenov, 1894 from Turkey and a key to the females (Coleoptera Meloidae)

**DOI:** 10.3897/zookeys.625.9545

**Published:** 2016-10-19

**Authors:** Marco A. Bologna, Andrea Di Giulio

**Affiliations:** 1 Dipartimento di Scienze, Università Roma Tre, viale G. Marconi, 446–00146 Roma, Italy

**Keywords:** Anatolia, key, new species, taxonomy, Teratolytta krejciki sp. n.

## Abstract

*Teratolytta krejciki* **sp. n.** from Munzur range (E Turkey) and a female belonging to a possible new species from the eastern Pontus range (N Turkey) are described and figured. New records of *Teratolytta gentilis* from southern Turkey are also provided. Difficulties to recognize females of this genus are discussed and a tentative key to the females of all species is proposed with the addition of a photographic plate.

## Introduction

The blister beetle genus *Teratolytta* Semenov, 1894 was revised by Bologna and Di Giulio (2006) who described four new species and the first instar larva (never studied in this genus), synthesized the information on ecology and ethology, included a key to males, proposed some taxonomic changes and a classification of the genus divided in two sections with five groups of species. Since this study only a faunistic and ecological contribution on *Teratolytta kulzeri* from Turkey has been published (Kemal and Koçak 2011). Females of this genus are extremely difficult to identify if they are not associated with males, which is why the key of the mentioned revision was based on the males only.

The genus is biogeographically centered in the Near and Middle East and shows the richest diversity in the Anatolian peninsula, where 10 out of 17 species occur. Just ten years after the revision of the genus a new species from eastern Turkey is described and a possible new species from northern Turkey is briefly discussed and figured in this work. These novelties increase the diversity of this genus in Anatolia.

Aims of this paper are: a) to describe and figure *Teratolytta krejciki* sp. n.; b) to briefly describe and figure a possible new species from eastern Pontus; c) to publish new records for *Teratolytta gentilis*; d) to provide a tentative key to females of the genus, enriched by a photographic synoptic table.

## Results

### Teratolytta krejciki sp. n.

Taxon classificationAnimaliaColeopteraMeloidae

http://zoobank.org/FD38A467-B10F-4582-BE20-A53200E6568C

Figs 1 , 2 , 3

#### Type material.

Holotype ♂ (S. Krejcik collection), labelled “26–27.6.2009; TR; Turkey, Pülümür, 2–7 km NW Pülümür, T. Tichny; 1550-2100 m; Tunceli”. A second label specifies “*Teratolytta* sp. det. Stanislav Krejcik 2011-2”. The holotype lacks the last right protarsomere.

#### Type locality.

“2–7 km NW Pülümür, T. Tichny; 1550–2100 m”. Pülümür is a small village of the Tunceli vilayet (province), in the eastern Turkey, placed at base of the southern slope of the Pülümür geçidi (pass), along the Munzur dağları (range). This locality is characterized by subtermophilic pastures and sparse woodlands.

The single specimen was collected early in the morning in a sparsely vegetated flood zone just north of Pülümür village. Although this locality has a very rich fauna of blister beetles and we collected about 30 species of meloids, other attempts to collect further specimens of *Teratolytta krejciki* in the same area during subsequent years (May, June) were not successful.

#### Diagnosis.

**Figure 1.**
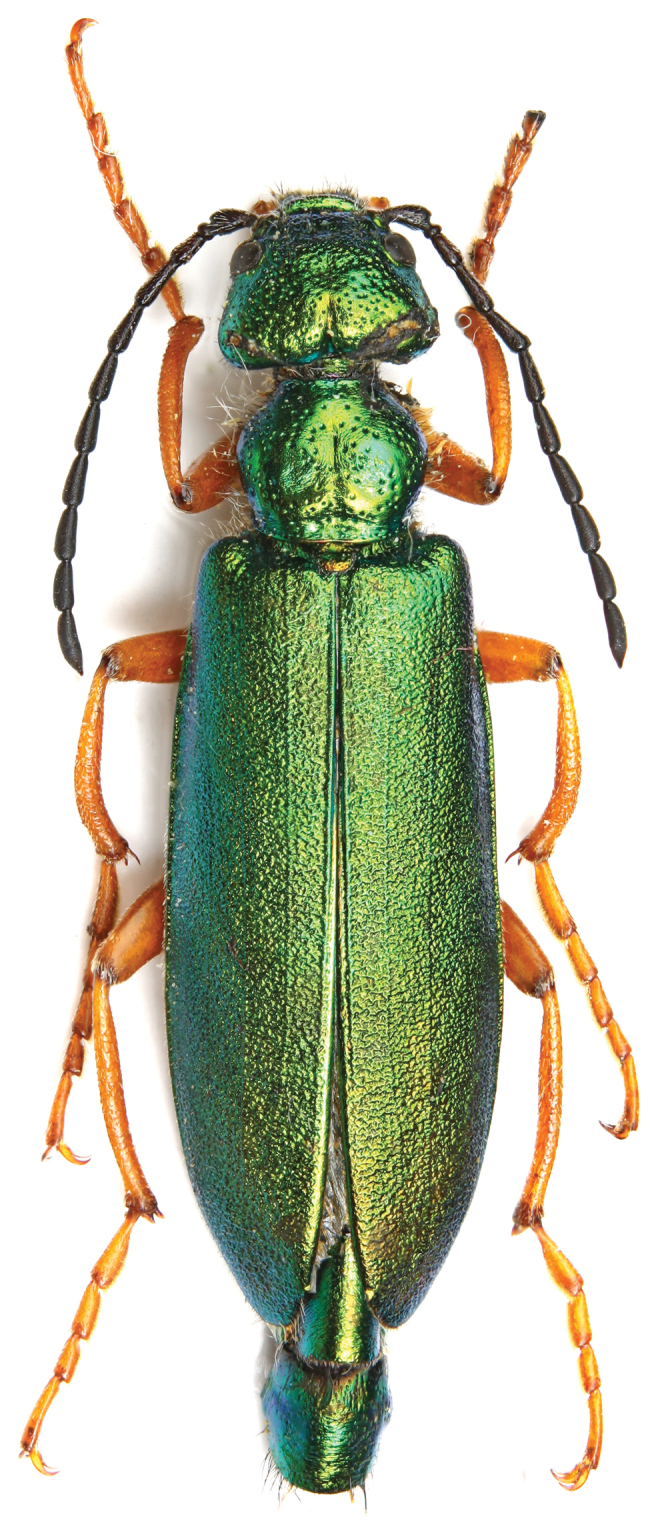
*Teratolytta krejciki* sp. n., habitus, male, dorsal view.

**Figures 2.**
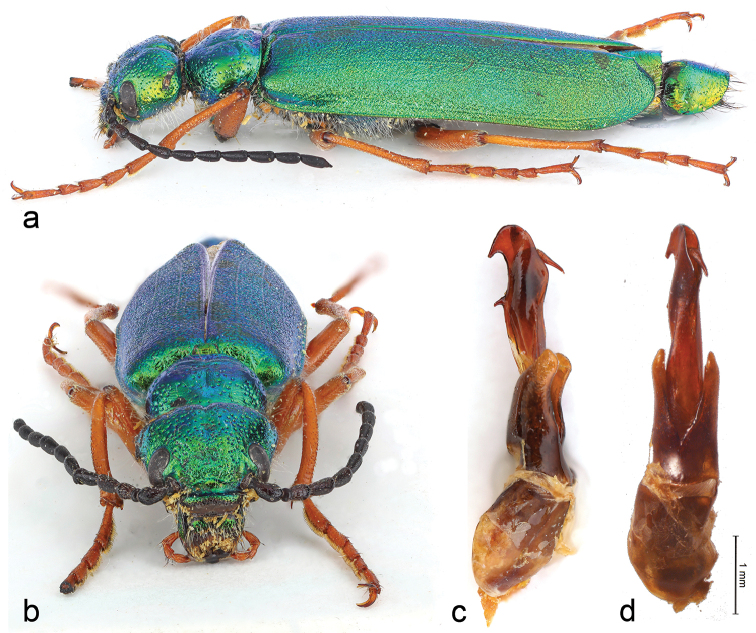
*Teratolytta krejciki* sp. n., male: **a** habitus lateral view **b** head, frontal view **c** tegmen and aedeagus, lateral view **d** tegmen and aedeagus, ventral view.

Large sized and slender species (22 mm) (Figs 1, 2a) belonging to the Section I as defined by Bologna and Di Giulio (2006). Body monochromatic metallic green, but legs red with metallic green coxa and black trochanter. Setation short, black on sides of pronotum and head, ventrally long and white. Head puncturation scattered, surface shiny. Sexual dimorphism not evaluable, female unknown. Male mesotibia not modified at apex but deeply curved on the apical half of inner side, and with micro-tubercles on external side. Male mesotarsomere I not modified and without modified black setae. Male metatrochanters triangularly elongate without modified apical setae; pro- and mesotibiae with two apical spurs, outer metatibial spur very large; protibiae only slightly curved. Parameres with robust apical lobes; aedeagus with two apical hooks greatly distanced to each other; endophallic hook slightly curved at apex.

#### Description.

**Figures 3.**
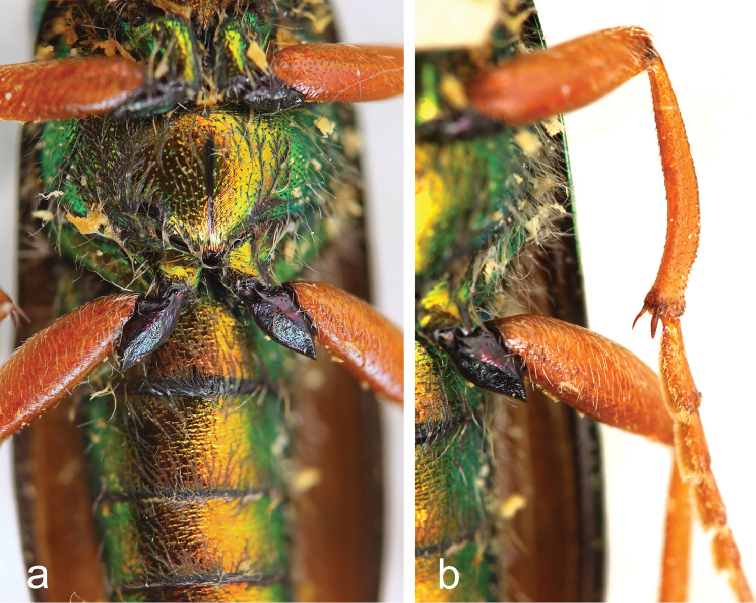
*Teratolytta krejciki* sp. n., male: **a** metasternum and metatrochanters **b** mesotibiae and mesotarsomeres.

*Body* (coxae included) shiny metallic green (Figs 1, 2a–b), abdomen metallic cupreous with posterior margin of sterna strictly black (Fig. 3a) and last two urites metallic green; maxillary palpi orange-red as well as legs, but coxae metallic green and trochanters black; mouthparts black, antennae subopaque black. Setation dorsally very short, particularly on elytra, black and slightly longer on the pronotum sides, genae and mouthparts. Modified setae of male last abdominal urite black. Body length (apex of mandibles to apex of elytra) 21.70 mm; head maximum width 3.74 mm; pronotum length 2.77 mm, width 3.25 mm; elytral greatest width at posterior third 5.46 mm. *Head* short subtrapezoidal (Fig. 2b), distinctly wider than long, maximum width at temples; sides of head obliquely narrowed from base to eyes; frons transversely depressed before the suture, convex in the middle and slightly depressed at level of the posterior margin of eyes; mandibles short, robust and curved; temples convex without postocular depression; clypeus convex; labrum slightly depressed and slightly emarginated at fore margin, with moderately deep scattered punctures, surface between punctures shiny; frontal suture almost straight; maxillary and labial palpomeres slender; last maxillary palpomeres longer than penultimate; antennae extending to basal third of elytra (Fig. 1, 2a); antennomere I about twice as long as II, subequal to III; III–X elongate, cylindrical; III slightly longer than the following; XI 1.5 times as long as X, cylindrical, narrowing in the apical third; antennomeres I-III with longer black setae. *Pronotum* (Fig. 1) shortly transverse, almost hexagonal, maximal width at middle, wider than long slightly depressed longitudinally in middle, slightly depressed transversally along the base; pronotal punctures as on head or slightly sparser laterally. Scutellum wide, subquadrate, with round and slightly depressed apex. Elytra elongate, feebly convex, narrowly rounded at apex, with vague traces of venation, uniformly rugose, setation extremely short. *Metasternum* without tubercles (Fig. 3a). Tibiae of all legs with two spurs, both slender and pointed on pro- and mesotibiae (Fig. 3b); spurs of metatibiae robust, the inner pointed, the outer very large, subtruncate apically; male tibiae of all legs cylindrical, not modified at apex, with simple setation and with spiniform and obtuse mixed tubercles on external side, mesotibiae without supplementary spine-like brush of setae, greatly curved on inner side in the posterior half (Fig. 3b), metatibiae slightly curved on inner side; male mesotarsomere not modified (Fig. 3b), tarsomere II not modified, with regular setae; male metatrochanters simple, triangularly elongate without modified apical setae (Fig. 3a). Last visible sternite of male abdomen emarginated, with modified apical setae on both sides, shorter than the entire sternite. *Parameres* (Fig. 2c–d) robust and with robust apical lobes; aedeagus with two apical hooks, distinctly distanced, different in shape and size, distal one smaller than proximal one (Fig. 2c); endophallus hook straight, acutely and shortly curved apically.

#### Etymology.

The new species is named after Stanislav (Standa) Krejčík, an active Czeck entomologist and excellent photographer, very interested in blister beetles, who, after recognizing the novelty of this *Teratolytta*, kindly sent us the single specimen of this new species with some nice photos (Figs 1, 2, 3a).

#### Taxonomic remarks.

According to Bologna and Di Giulio (2006) two sections are recognized in the genus, based on the absence (section I), or presence (section II) of two tubercles on metasternum, just posterior to the base of the middle legs. *Teratolytta krejciki* clearly belongs to section I due to certain male symplesiomorphic features such as lack of tubercles on metasternum and presence of two spurs on pro- and mesotibiae. This species is immediately distinct from *Teratolytta gentilis* group because mesotibiae and mesotarsomeres are not modified, and from *Teratolytta pilosella* group by lack of depression on males genae. It differs from *Teratolytta klapperichi* group because of the presence of two pro- and mesotibial slender spurs and two big aedeagal hooks, and from *Teratolytta kaszabi* group because of the distanced aedeagal hooks.

The new species does not shows any peculiar modification on male mesotibiae, mesotarsomeres, metathorax, which are present in several *Teratolytta* species (Bologna and Di Giulio 2006), a condition similar to *Teratolytta carlae*, but this last species greatly differs from *Teratolytta krejciki* at least because of the following features: smaller size and stout body shape; body colour, metallic blue or metallic green with a cupreous stripe; aedeagal hooks both at apex and smaller.

### Teratolytta

Taxon classificationAnimaliaColeopteraMeloidae

sp. A (possible new species related to Teratolytta carlae Bologna, 2006)

Fig. 4a

#### Material examined.

1 ♀ (M.A. Bologna collection), labelled “Turkey 12. Gümüṣhane, 14 km N Kelkit, 6 km N Ülüpinar, 40.1533N -39.2847E, 1850–2150 m, 28.6.2013, M.A. Bologna, P. Rapuzzi & P. Audisio” (by pitfall traps positioned 30 days before).

This locality is placed on the southern slope of the eastern Pontus range, in a submesophilic mountain habitat, characterized by a mosaic of *Quercus* woodlands and pastures, with *Crataegus* and other Rosaceae in the ecotonal borders.

#### Diagnostic characters and comparative analysis.

**Figure 4.**
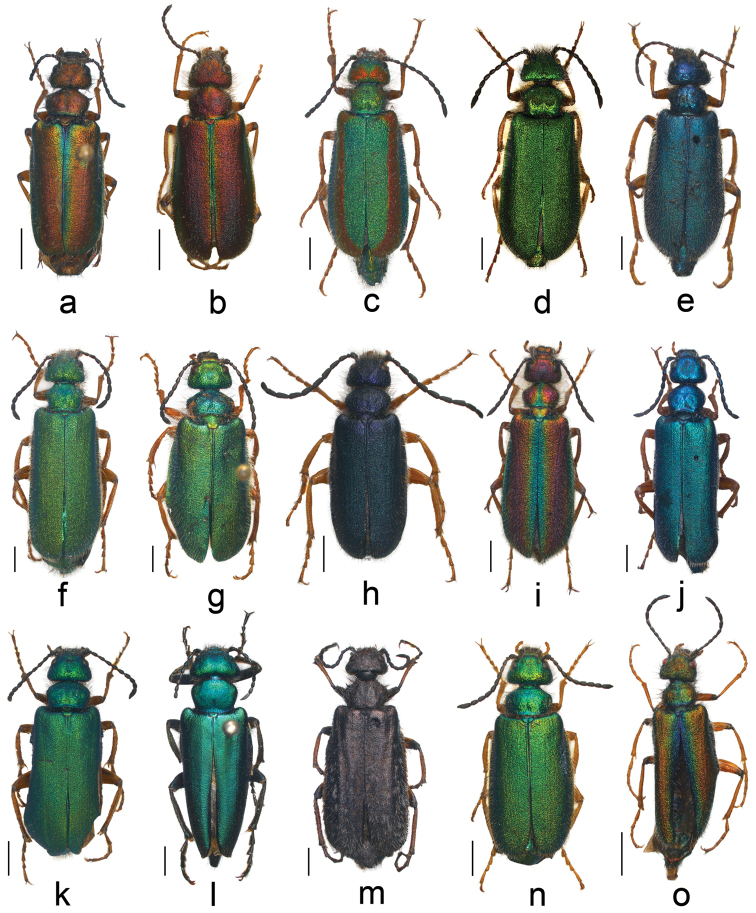
Females of the genus *Teratolytta*: **a** *Teratolytta* sp. A (sp. n. ?) **b** *Teratolytta carlae* **c** *Teratolytta dives*, striped phenotype **d** *Teratolytta dives*, unicoloured phenotype **e** *Teratolytta eylandti* **f** *Teratolytta flavipes* **g** *Teratolytta gentilis*, typical phenotype **h** *Teratolytta gentilis*, blue phenotype **i** *Teratolytta kaszabi* **j** *Teratolytta klapperichi* **k** *Teratolytta monticola* **l** *Teratolytta optabilis* **m** *Teratolytta pilosella* **n** *Teratolytta taurica* **o** *Teratolytta vanensis*. Scale bars 2 mm.

Middle sized species (Fig. 4a), length 17 mm; body integument metallic green but head, pronotum (except base, which is green), one very wide longitudinal stripe along each elytron, meso- and metathoracic sternites and abdominal sternites metallic cupreous; clypeus, labrum, mandibles and antennae black; maxillary and labial palpomeres respectively light and dark red-orange, last maxillary palpomere black at apex; legs red-orange, but coxae, trochanters, apex of femurs and base of tibiae black.

*Head* transversely trapezoidal, with dense and deep punctures, slightly depressed in front; black elongate setae on temples. Antennae short (Fig. 4a), extending to or a little beyond the base of pronotum; antennomeres short, particularly V-VII. *Pronotum* transversely subexagonal (Fig. 4a), maximal width just anterior to the middle, sides subrounded in the basal half; in the middle with a slight mid-longitudinal depression; punctures slightly sparser than on head. Scutellum subrectangular, rounded at apex, with long black setae particularly on sides. Elytra weakly convex only on the basal third, subrugose, with long whitish setae, denser posteriorly and on lateral margins. Ventral side with dense and long whitish setation. *Legs* not modified; tibiae straight; all legs with two tibial spurs, fore and middle slender, posterior spurs spatuliform, external spur wider; claws distinctly curved.

The single female does not correspond to any described species and possibly represents a new undescribed species. We prefer not to describe it and wait for the discovery of the male.

Six other species in both Section I and II have a cupreous longitudinal stripe on the elytra: (a) *Teratolytta carlae* Bologna, 2006 (Fig. 4b); *Teratolytta kaszabi* Kryzhanovskij, 1959 (Fig. 4i); *Teratolytta regina* Kaszab, 1958; (b) *Teratolytta dives* (Brullé, 1832) (Fig. 4c), *Teratolytta tricolor* (Haag-Rutenberg, 1880), *Teratolytta vanensis* Kaszab, 1968 (Fig. 4o). Species A differs from the species in Section II because it has a narrower cupreous elytral stripe, metallic green head and pronotum, distinctly longer antennae, extending to the fore third of elytra, more slender and elongate antennomeres V-VII. Moreover in *Teratolytta dives* and *Teratolytta tricolor* the pronotum is wider in front, slightly concave, not rounded with whitish setae and head and pronotum punctures are bigger. Due to the more expanded black coloration of knees and black setation on pronotum and head, the probable new species is more similar to *Teratolytta vanensis*, which differs by its notably smaller size, narrower pronotum and distinctly longer antennae.

Comparing this new *Teratolytta* species with other striped species of Section I, *Teratolytta kaszabi* and *Teratolytta regina*, have slightly longer antennae, similar body size and both are distributed in Central Asia. Moreover, *Teratolytta kaszabi* has red unicolour knees and more hexagonal and narrower pronotum. The probable new species is similar to *Teratolytta carlae* in colour of setae on head and pronotum, colour of knees and body and the length of antennae, but differs by smaller size, less slender body, and slightly widened posterior portion of elytra . The striped form in *Teratolytta carlae* is a variant and other specimens are uniformly blue.

### Teratolytta gentilis

Taxon classificationAnimaliaColeopteraMeloidae

(Frivaldszky, 1877)

Fig. 4g–h

#### New records.

(Eğirdir) Yukangökdere, 37.42964N, -30.49899E, Kasnak forest, window-trap 17, Hollow *Quercus*, 17.5.2007, N. Jonsson & M. Avci (Konya), Güneyyurt Km 2 SE, 1.6.2011, F, Angelini (Antalya) Akseki, 1500 m, 22.5.1997, P. Rapuzzi. All specimens are housed at the M. A. Bologna collection (University Roma Tre).

These new records improve the distribution of this Anatolian species in southern Turkey and confirm a doubtful record from Antalya province cited by Bologna and Di Giulio (2006).

### Key to the females of the genus *Teratolytta* (see Fig. 4)

In the revision of the genus (Bologna and Di Giulio 2006), the identification key was provided only for males due to the difficulties in detecting distinctive characters of females. In the following key we tentatively distinguish the females of all species except *Teratolytta dvoraki* and *Teratolytta krejciki*, which are still unknown. The key is mostly based on the colour of body parts, because we did not find diagnostic characters such as in males. In order to help with the identification of females, a colour plate (Fig. 4) of most of species is provided except *Teratolytta dvoraki*, *Teratolytta krejciki*, and *Teratolytta regina* and *Teratolytta tricolor*, which were unavailable.

**Table:** 

1	Elytra metallic green with a longitudinal, more or less widened cupreous stripe	**2**
–	Elytra unicolourous	**8**
2	Antennae distinctly extending to the basal third of elytra; cupreous stripe narrow (Fig. 4c, o); pronotum metallic green; middle antennomeres (V–VIII) slender cylindrical	**3**
–	Antennae extending to the base of pronotum or only slightly beyond; cupreous stripe wide (Fig. 4b, i); pronotum cupreous; middle antennomeres (V–VIII) obtusely cylindrical	**5**
3	Pronotum maximal width in the apical third, distinctly subhexagonal, sides slightly concave at basal half	**4**
–	Pronotum maximal width at middle, not subhexagonal, sides slightly convex externally at basal half	***Teratolytta vanensis* Kaszab, 1968**
4	Species distributed in Balkans an Anatolia	***Teratolytta dives* (Brullé, 1832)** (pars)
–	Species distributed in NE Iran and SW Turkmenistan	***Teratolytta tricolor* (Haag-Rutenberg, 1880)**
5	Apex of femur and base of tibia uniformly red-orange	***Teratolytta kaszabi* Kryzhanovskij, 1959**
–	Apex of femur and base of tibia more or less extensively black	**6**
6	Antennae distinctly extending beyond the base of pronotum, antennae elongate. Species distributed in Afghanistan	***Teratolytta regina* Kaszab, 1958**
–	Antennae short (Fig. 4a), extending to or a little beyond the base of pronotum, antennomeres short. Species distributed in Turkey	**7**
7	Body stout, posterior third of elytra slightly widened	***Teratolytta carlae* Bologna, 2006** (pars)
–	Body slender, the posterior third of elytra parallel	***Teratolytta* sp. A**
8	Head, pronotum and elytra dark bronze	***Teratolytta pilosella* (Solsky, 1881)**
–	Body colour different	**9**
9	Head, pronotum and elytra dark metallic blue	**10**
–	Head, pronotum and elytra metallic green or green-bluish, not uniformly dark blue	**12**
10	Apex of femur and base of tibia uniformly red-orange	***Teratolytta gentilis* (Frivaldszky, 1877)** (pars)
–	Apex of femur and base of tibia black	**11**
11	Head and pronotum subopaque; antennomeres III-VI short	***Teratolytta carlae* Bologna, 2006** (pars)
–	Head and pronotum shiny; antennomeres III-VI elongate	***Teratolytta eylandti* Semenow, 1894**
12	Legs black; dorsal body surface sparsely micropunctate, subopaque	***Teratolytta optabilis* (Falderman, 1832)**
–	Legs totally or partially red-orange; dorsal body surface with middle sized punctures, more or less scattered, shiny	**13**
13	Apex of femur and base of tibia black; pronotum transverse, subhexagonal, slightly wider than head at temples	***Teratolytta monticola* Bologna, 2006**
–	Apex of femur and base of tibia red-orange or base of tibia vaguely dark; pronotum variously shaped	**14**
14	Pronotum not transverse, slightly narrower than head width at temples, sides subrounded or parallel, or slightly angulate	**15**
–	Pronotum clearly transverse, slightly wider than head at temples, notably subtrapezoidal, sides distinctly angulate	**18**
15	Head distinctly depressed in the middle; middle antennomeres with vague green-blue metallic reflections. Species distributed in Afghanistan	***Teratolytta klapperichi* Kaszab, 1958**
–	Head not distinctly depressed in the middle; antennomeres black, subopaque, except for black metallic reflection of antennomere I. Species distributed in Turkey and Syria	**16**
16	Pronotum with sparse punctation; pronotal sides arcuate subrounded; dorsal surface greenish-blue; head and pronotal setae mostly black, mixed with short whitish setae, dorsal elytral setae black	***Teratolytta kulzeri* Kaszab, 1958**
–	Pronotum with dense punctuation and in some areas subrugose; pronotal sides subparallel or slightly angulate; dorsal surface distinctly green; head and pronotal setae white and long, dorsal elytral setae white	**17**
17	Hind trochanter red-orange	***Teratolytta senilis* (Abeille de Perrin, 1895)**
–	Hind trochanter black	***Teratolytta flavipes* (Mulsant & Rey, 1858) and *Teratolytta dives* (Brullé, 1832)** (pars)
18	Pronotum with sparse punctation, basal margin straight; antennomere I black; dorsal elytral setae sparse; mesotibiae slightly curved	***Teratolytta taurica* Bologna, 2006**
–	Pronotum with dense punctuation, basal margin slightly sinuate in the middle; antennomere I with metallic violet-cupreous reflections; dorsal elytral setae denser; mesotibiae distinctly curved	***Teratolytta gentilis* (Frivaldszky, 1877)** (pars)

## Supplementary Material

XML Treatment for Teratolytta krejciki

XML Treatment for Teratolytta

XML Treatment for Teratolytta gentilis
